# Characterization of intracranial atherosclerotic stenosis using high‐resolution MRI study – rationale and design

**DOI:** 10.1002/brb3.397

**Published:** 2015-09-25

**Authors:** Tanya N. Turan, Todd LeMatty, Renee Martin, Marc I. Chimowitz, Zoran Rumboldt, M. Vittoria Spampinato, Seth Stalcup, Robert J. Adams, Truman Brown

**Affiliations:** ^1^ Department of NeurologyMedical University of South CarolinaCharlestonSouth Carolina; ^2^ Department of Public Health SciencesMedical University of South CarolinaCharlestonSouth Carolina; ^3^ Department of Radiology and Radiological SciencesMedical University of South CarolinaCharlestonSouth Carolina

**Keywords:** Atherosclerotic plaque pathology, high‐resolution MRI, intracranial atherosclerosis, stroke

## Abstract

**Background:**

Intracranial atherosclerosis is a leading cause of stroke, but little is known about the composition of the intracranial atherosclerotic lesion and how intracranial plaque morphology is related to the risk of stroke. High‐resolution magnetic resonance imaging (HR MRI) has been used in patients with extracranial carotid atherosclerosis as an in vivo tool to identify, with high‐interrater agreement, histologically defined plaque components (i.e., intraplaque hemorrhage, fibrous cap, and lipid core), which have been shown to be predictors of recurrent stroke. With careful imaging the components of atherosclerotic plaque can be visualized in the intracranial arteries using HR MRI, but there are no reports of reproducibility or interrater reliability.

**Methods/Study design:**

The Characterization of intracranial atherosclerotic stenosis using high‐resolution MRI (CHIASM) study is a single‐center NIH‐funded prospective observational study, to (1) demonstrate high ‐interrater agreement for identifying intracranial plaque components on HR MRI, (2) determine the frequency of these components in symptomatic versus asymptomatic plaques, and (3) estimate the 1‐year rate of stroke in the territory of high‐risk plaque components. CHIASM will recruit 90 patients with 50–99% intracranial atherosclerosis to undergo HRMRI of the intracranial artery plaque at enrollment and 1‐year follow‐up. Both symptomatic and asymptomatic subjects will be recruited.

**Conclusion:**

Determination of good interrater reliability is an important first step in the development of HR MRI as a tool to predict risk in patients with intracranial atherosclerosis. This study will inform the design of future multicenter studies to determine the prevalence and prognosis of intracranial atherosclerotic plaque components. Such studies could lead to new understanding of the pathophysiological mechanisms of cerebral ischemia in patients with atherosclerotic intracranial stenosis, improvements in risk stratification, and potentially to new treatments of this common and serious disease.

## Introduction

Intracranial atherosclerotic disease causes 30–50% of strokes in Asia (Wong 2006) and 8–10% of strokes in North America (Sacco et al. 1995), making it one of the most common causes of stroke worldwide (Gorelick et al. 2008). In the US, approximately 70,000 strokes per year caused by intracranial atherosclerosis cost $9 billion over the lifetime of these patients (lifetime cost of stroke per patient $140,000) (Sacco et al. 1995; Roger et al. 2012). Symptomatic intracranial arterial stenosis (ICAS) is also one of the highest risk conditions resulting in recurrent ischemic stroke. Despite the enormous impact of ICAS worldwide, there have been limited studies on its biology and the characteristics of its plaques that are associated with a high risk of stroke. This is due to several reasons. First, unlike the case of extracranial carotid stenosis, it is not possible to obtain pathological specimens of ICAS in living patients. Second, there are few postmortem studies of ICAS. Finally, there are no animal models that can replicate the pathophysiology of ICAS. This makes the recent development of in vivo tools for studying ICAS plaque features a very significant breakthrough and it is very likely to improve our ability to predict stroke risk and monitor response to stroke prevention treatments.

## Rationale

### HR MRI will improve stroke prevention

Demonstrating that HR MRI‐defined plaque components are important predictors of recurrent stroke in patients with intracranial stenosis could lead to changes in the selection of patients for future clinical trials that evaluate new treatments for patients with intracranial stenosis. For example, clinical trials that previously would have used high‐risk predictors, such as severe stenosis, might in the future include patients with plaque predictors of recurrent stroke (e.g., patients with 50–69% stenosis and high‐risk plaque features). Establishing that plaque components are predictors of intracranial atherosclerotic progression could also lead to new strategies for clinical trial design. Rather than focus on clinical events, clinical trials could evaluate new therapies targeted at preventing progression of intracranial atherosclerosis in patients with high‐risk plaque components, as has been done in clinical trials to evaluate the effects of rosuvastatin on plaque progression in patients with extracranial carotid atherosclerosis and HR MRI‐defined lipid core (Underhill et al. 2008)^.^ Similarly, the presence or absence of established high‐risk HR MRI intracranial plaque components could also be used as a surrogate endpoint in clinical trials to evaluate the treatments for intracranial stenosis, instead of waiting for clinical endpoints, such as recurrent stroke, which may take years to become apparent. This strategy has already been employed in trials evaluating statins in patients with aortic atherosclerosis (Yonemura et al. 2005) and extracranial carotid atherosclerosis (Corti et al. 2002). In summary, the development of HR MRI as a clinical tool for risk stratification, marker for disease activity, and a research tool to study intracranial atherosclerotic plaque development and progression could lead to new stroke prevention treatments, which could then result in a significant decrease in the economic and social burden caused by stroke in the US and worldwide due to intracranial atherosclerosis.

### HR MRI will lead to new understanding of stroke pathophysiology in intracranial stenosis

In addition to improvements in stroke prevention, the development of an in vivo imaging tool such as HR MRI to evaluate the plaque components in patients with intracranial atherosclerosis could also lead to increased understanding of how intracranial atherosclerosis results in cerebral ischemia. Plaque rupture is the most common mechanism of ischemia in coronary arteries and features of “vulnerable plaque” (or plaque prone to rupture) are well described (Stary et al. 1995). However, it is not clear what role plaque rupture has in causing ischemia in intracranial atherosclerosis or which plaque features may make an intracranial plaque “vulnerable”. The small postmortem studies that have evaluated whether plaque rupture plays an important role in causing stroke in intracranial atherosclerosis have produced inconclusive data, with one small series showing a minority of patients had plaque rupture (Ogata et al. 1994) and another study showing that a majority had plaque rupture (Sadoshima et al. 1979). The plaque composition has also been studied in a postmortem series, which found that intracranial plaques associated with infarct (*n *= 18) had (1) a higher area of lipid core, (2) a higher frequency of neovasculature and thrombus, (3) a trend toward a higher frequency of intraplaque hemorrhage, but (4) no difference in fibrous cap thickness compared to the plaques not associated with infarct (*n *= 38) (Chen et al. 2008). These findings show that intracranial plaque components may differ between symptomatic and asymptomatic plaques. However, using postmortem series to understand intracranial plaque pathology in stroke patients has important limitations as the vast majority of stroke patients do not die acutely from their strokes. As such, the cerebrovascular pathological findings at autopsy may not reflect the intracranial plaque morphology associated with most acute cerebral ischemic events. In order to study the intracranial plaque morphology associated with acute cerebral ischemic events, an in vivo imaging technique, such as HR MRI, is needed.

High‐resolution magnetic resonance imaging might also be able to explain some cryptogenic ischemic strokes without previously identifiable causes, which make up approximately 30% of the strokes. Currently, atherosclerotic narrowing greater than 50% is considered a likely cause of stroke (Warfarin‐Aspirin Symptomatic Intracranial Disease Trial Investigators, 2003), but HR MRI might be able to detect early atherosclerotic “positive remodeling” (i.e., compensatory enlargement of both the outer wall of the vessel as well as the lumen) prior to the development of flow‐limiting stenosis (Hermiller et al. 1993) or detect nonstenotic plaque that could result in local penetrating artery occlusion (Klein et al. 2010). Evidence from histopathological and clinical studies suggests that positive remodeling is associated with plaque vulnerability and ischemic events in coronary arteries (Burke et al. 2002), but has not been as well studied in intracranial arteries.

In summary, establishing that HR MRI can reliably characterize intracranial atherosclerotic plaque components and that plaque composition can predict recurrent stroke could result in (1) a clinical tool for risk stratification, (2) a marker for disease activity, and (3) a research tool to further study atherosclerotic plaque development and progression and mechanisms of ischemia in intracranial stenosis.

## Study Design

### Overview

This is an NIH‐funded single‐center prospective observational study of subjects with intracranial atherosclerotic plaque that aims to establish HR MRI as a reliable tool to identify intracranial atherosclerotic plaque components and to obtain preliminary data on the prognosis of the proposed high‐risk plaque components. Establishing the reliability of HR MRI will be done by determining the interrater agreement of HR MRI to visualize the intracranial plaque components.

### Study hypotheses and aims

The primary hypothesis is that in patients with intracranial atherosclerotic stenosis (50–99%) of a major intracranial artery, the interrater agreement via a kappa statistic of two readers for identifying each of the plaque components (intraplaque hemorrhage, thin or ruptured fibrous cap, higher lipid core scores) on HR MRI will be greater than 0.80 (excellent agreement). Other aims are to (1) compare the frequency of high‐risk plaque features between asymptomatic and symptomatic intracranial plaques; (2) estimate the 1‐year rate of symptomatic or clinically silent ischemic strokes in the territory of intracranial plaques with at least one high‐risk component versus plaques with no high‐risk components; (3) estimate the change in the frequency of plaque components and the frequency of the plaque progression or regression from baseline to 1 year; and (4) explore the association between plaque progression and control of vascular risk factors.

### Eligibility criteria

**Figure 1:**
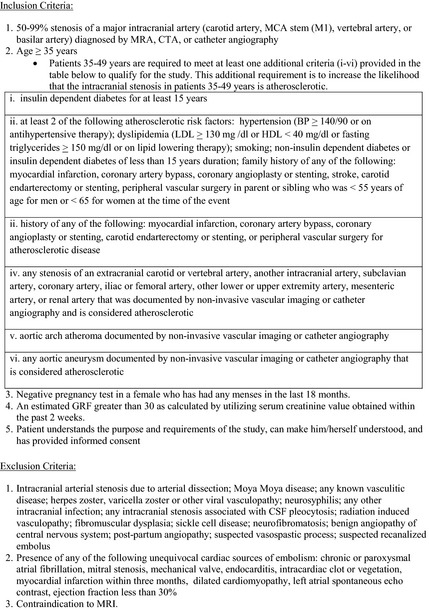
Inclusion and exclusion criteria.

Patients with intracranial atherosclerosis will be recruited for this study. We will prospectively study 100 intracranial atherosclerotic plaques in vivo (50 symptomatic plaques, 50 asymptomatic plaques). Patients will be identified for this study based on the results of vascular imaging studies (MRA, CTA, or catheter angiography) done as part of routine care. The inclusion and exclusion criteria are provided in Figure 1. Plaques will be considered symptomatic if the patient has had a TIA or stroke in the same vascular territory within 30 days prior to the enrollment and no other cause of TIA or stroke is identified despite a full diagnostic evaluation. This is the same definition used in the multi‐center clinical trials of intracranial stenosis (Chimowitz et al. 2005; Chimowitz 2008). The plaques will be considered asymptomatic if the patient has not had a TIA or stroke in the same vascular territory within 90 days prior to the enrollment. Asymptomatic plaques will be identified in patients who undergo vascular imaging for stroke in another vascular territory (i.e., pontine stroke and an asymptomatic MCA stenosis) or patients with vascular imaging done for other reasons (i.e., headache or aneurysm).

Approximately 20% of symptomatic subjects are expected to also have an asymptomatic stenosis, based on the frequency of asymptomatic intracranial stenosis observed in the WASID trial (Nahab et al. 2008), and thus will contribute to both groups. Therefore, it is expected that of the 50 patients with a symptomatic plaque, 10 will also have an asymptomatic plaque, resulting in the need to recruit only 40 subjects with an isolated asymptomatic plaque (total number of subjects is 90 patients over 2–4 years or 30 patients per year).

### Baseline study assessments and data collection

#### Clinical assessments

Health information related to current medication use, prior stroke or TIA, NIH Stroke Scale, other medical comorbidities, and risk factor information (e.g., blood pressure, cholesterol, and HgA1c values) will be collected at baseline, and 6 and 12 months after the enrollment. Information is obtained from the patient and the medical record.

#### MR imaging

The HR MRI sequences will be performed on a 3T MRI with a 32‐channel head coil with a total scan time of approximately 40 min. To localize the artery of interest, 3D TOF angiography of the Circle of Willis will be performed, followed by the following single‐slab 3D acquisitions:

1. T1‐weighted: TR/TE 458/16, FA 180°, matrix 320 × 320, 11 slices, thickness 1.2 mm, and FOV 128 mm
2. T2‐weighted: TR/TE 1500/66, FA 180°, matrix 256 × 256, 11 slices, thickness 1.2 mm, and FOV 104 mm
3. Fluid attenuated inversion recovery (FLAIR): TR/TE 2500/14, inversion time (TI) 1069, FA 140°, matrix 256 × 197, 11 slices, thickness 1.2 mm, and FOV 100 mm
4. T1‐weighted: TR/TE 458/16, FA 180°, matrix 320 × 320, 11 slices, thickness 1.2 mm, FOV 128 mm, and post gadolinium (Multihance)

Whole brain FLAIR images will also be obtained to assess for ischemic lesions at baseline and 1‐year follow‐up.

### Follow‐up study assessments and schedule of visits

Study patients will undergo a clinical follow‐up visit at 6 months (by phone if necessary) and repeat MRI and clinical visit at 1 year after the study enrollment. The repeat MRI sequence is identical to the enrollment MRI and care is taken to ensure an identical positioning during acquisition.

### Classification of plaque components using HR MRI

Comparative studies of HR MRI and pathological carotid endarterectomy specimens have shown that HR MRI can identify the plaque components intraplaque hemorrhage (Moody et al. 2003; Chu et al. 2004), fibrous cap (Hatsukami et al. 2000; Mitsumori et al. 2003), and lipid core (Saam et al. 2005) with good sensitivity (81–90%) and specificity (74–90%). The use of T1 MPRAGE (or “black blood”) sequences in particular have demonstrated pathologically verified intraplaque hemorrhage (Moody et al. 2003; Chu et al. 2004; U‐King‐Im et al. 2008; Findeiss et al. 2010) and can accurately differentiate lipid core from intraplaque hemorrhage (Findeiss et al. 2010). The addition of gadolinium contrast has also resulted in improved methods for the detection of ruptured fibrous cap, as several studies have demonstrated that the enhancement within the plaque correlates with fibrous cap rupture in histological specimens (Wasserman et al. 2002; Yuan et al. 2002a; Kawahara et al. 2007; Kerwin et al. 2009). Using these definitions, HR MRI of extracranial carotid atherosclerosis has good interrater reliability. Several carotid atherosclerosis studies have demonstrated that the techniques for measurement of the high‐risk plaque features intraplaque hemorrhage (Altaf et al. 2008), fibrous cap (Trivedi et al. 2004a,b), and lipid core (Trivedi et al. 2004a,b; Saam et al. 2005) were reliably detectable by readers with Kappa scores >0.80 (excellent agreement). Several prospective studies have shown that HR MRI‐defined plaque component definitions utilized in this study [intraplaque hemorrhage (Takaya et al. 2006; Altaf et al. 2007a, 2008; U‐King‐Im et al. 2008), thin or ruptured fibrous cap (Yuan et al. 2002b; Takaya et al. 2006; U‐King‐Im et al. 2008), and larger lipid core area (Takaya et al. 2006; U‐King‐Im et al. 2008)] are strongly associated with the occurrence of stroke or TIA in patients with extracranial carotid plaque. Therefore, we will use the definitions derived from these studies, as listed in Table 1 above.

**Table 1:** Classification of plaque components on HR MRI

Plaque feature	Signal characteristics	Classification
Intraplaque hemorrhage (Altaf et al. 2007b, 2008)	Bright T1 signal (precontrast)	IPH present: ≥150% of T1 signal of adjacent muscle or pons
		IPH absent: T1 signal is < 150% of adjacent muscle or pons
Fibrous cap (Trivedi et al. 2004a,b)	Band of T2 high signal adjacent to lumen OR Enhancement of plaque on T1 (postcontrast)	Thick cap: the cap can be visualized and measured on T2. Thin or ruptured cap: no cap visualized with smooth or rough surface of plaque on T2 OR a clear defect or ulceration within the fibrous cap on T2 OR enhancement of plaque on T1 (postcontrast)
Lipid Core (Trivedi et al. 2004a,b; Saam et al. 2005)	Iso‐ to hyperintense on T1 (precontrast) Hypointense to isointense on T2	Area of T1 signal (precontrast) identified as lipid core is manually traced and area calculated. Lipid core % area = area of lipid core ÷ plaque area Plaque area = area of entire vessel – area of lumen Lipid Core Score: 0 = no lipid core identified 1 = lipid core area < 25% 2 = lipid core area >25%

### HRMRI image interpretation

The images will be interpreted by two of three readers who are fellowship trained in Neuroradiology (MVS, ZR, and SS) and they will be blinded to the plaque classification (symptomatic vs. asymptomatic). To keep the readers blinded to the symptomatic status of the patients, they will be asked to view preselected images that are limited to showing the atherosclerotic plaques only (i.e., they will not be given brain MRI images). However, it is recognized that in a few subjects who have had an ischemic stroke in the territory of the intracranial plaque, the readers may inadvertently see evidence of prior stroke (e.g., pontine stroke adjacent to a basilar plaque). Note will be made of those cases to determine what, if any, impact they have on the final results. It is important to note, however, that the reader will always be blinded to the *timing* of the ischemic stroke because no diffusion‐weighted scans will be performed.

### Data management

Clinical, demographic, and scored imaging data will be recorded on Case Report Forms and then entered into a secure web‐based data capture application entitled “REDCap (Research Electronic Data Capture)” available through the South Carolina Clinical and Translational Research Center (SCTR) (Harris et al. 2009). The MRI images will be stored using the secure MUSC informatics management system, which consists of an integrated system of Linux workstations surrounding a central core of Linux servers.

### Sample size estimation

The sample size was estimated to be 50 symptomatic and 50 asymptomatic intracranial plaques based on the number of subjects for whom it was feasible to recruit during the 4‐year period. The power and effect size calculations were then performed to determine if there was sufficient power to test the primary hypothesis (determination of interrater reliability) given the number of subjects available. The expected frequency for each of the plaque components in intracranial atherosclerosis was extrapolated from other studies and our pilot data (see Table 2). An estimated Kappa (Fleiss 1981) statistic was used to assess the ability to measure the agreement between two raters for each of the plaque features given 100 plaques. The estimated Kappas are: intraplaque hemorrhage (expected frequency ~30%): with 5% discordance, kappa is between 0.830–0.932, with 10% discordance, kappa is between 0.693–0.832; thin or ruptured fibrous cap (Xu et al. 2009) and lipid core area (U‐King‐Im et al. 2008) > 25% (expected frequency ~50%): with 5% discordance, kappa is between 0.858–0.943, with 10% discordance, kappa is between 0.741–0.859. Based on the guidelines for the values of Kappa in describing agreement (Landis and Koch 1977), all of the estimated Kappa values calculated above are considered to be within an acceptable range (0.4–0.80 – fair to good agreement and >0.80 – excellent agreement).

**Table 2:** Frequency of plaque characteristics in patients with atherosclerotic stenosis

Characteristic	Location and degree stenosis, *n *= patients	Symptomatic	Asymptomatic	Diagnostic modality
Intraplaque hemorrhage	Extracranial carotid stenosis 50–99%, *n *= 40	46.5%	14%	HR MRI (U‐King‐Im et al. 2008)
	MCA stenosis >40%, *n *= 76	30%	15%	Pathology (Chen et al. 2008)
	MCA stenosis >70%, *n *= 109	19.6%	3.2%	HR MRI (Xu et al. 2012)
	Intracranial stenosis 50–99%, *n *= 20	33%	20%	MRI CHIASM pilot^a^
Thin or ruptured fibrous cap	Extracranial carotid stenosis 50–99%, *n *= 58	96%	60%	HR MRI (Yuan et al. 2002b)
	Extracranial Carotid stenosis 50–99%, *n *= 40	88.4%	49%	HR MRI (U‐King‐Im et al. 2008)
	MCA stenosis > 50%, *n* = 47	68%	32%	HR MRI (Xu et al. 2009)
	Intracranial stenosis 50–99%, *n *= 20	36%	33%	MRI CHIASM pilot^a^
Lipid area > 25% of plaque	Extracranial carotid stenosis 50–99%, *n *= 40	63.8%	28%	HR MRI (U‐King‐Im et al. 2008)
	Intracranial stenosis 50–99%^a^, *n *= 20	75%	60%	MRI CHIASM pilot^a^

MCA, middle cerebral artery; ICA, intracranial internal carotid artery.

a Unpublished data: Five patients with a symptomatic plaque also had an asymptomatic plaque (total plaques = 25): 14 MCA, 2 ICA, 4 Basilar, and 5 Vertebral; No. symptomatic plaques = 19, No. asymptomatic plaques = 6.

### Statistical analysis

For the primary analysis, a Kappa (Fleiss 1981) statistic will be used to quantify the magnitude of agreement between the two raters for each of the plaque components and a Kappa of > 0.8 will be considered an excellent interrater agreement. The interrater agreement for the detection of the plaque components is expected to be as high in the intracranial circulation as it has been reported in the extracranial carotid circulation. However, it is possible that at least one of the plaque components may be an unreliable measure. If a plaque component is not found to be a reliable measure, it will be excluded from the subsequent analyses.

To determine if there is sufficient power to test the hypothesis that the symptomatic plaques would have a higher frequency of high‐risk plaque components than the asymptomatic plaques and given the estimated number of subjects, an inflation factor was used to inflate the sample size to account for the correlation of the values within subjects because a single subject could have both symptomatic and asymptomatic plaque. The inflation factor quantity takes into account the reduction in precision of the estimates when replications within a cluster are correlated (Donner and Klar 2000). The inflation factor [1 + (*m*−1) *ρ*] was used, where m is the average number of units per cluster and *ρ* is the estimated intraclass correlation coefficient (ICC) for clustering. Due to the correlation of the values within subjects, the effective sample size (ESS) is smaller than the total sample size needed for the study. In particular, the ESS is

$$\text{ESS}=\frac{N}{\left[1+(m-1)\rho \right]}$$

To calculate the power for each plaque feature, the following assumptions have been made: (1) Assume alpha to be 0.017 (0.05/3) to account for the multiple outcomes (i.e., each of the three plaque features is significantly different between the two groups at a 0.05 level). This is the most conservative approach, but is justified. If one or more of the plaque features are deemed to have interrater agreement less than the prespecified criteria Specific for Aim 1 (kappa < 0.8), it will not be included as a primary outcome. This will result in preservation of the overall power for the study. (2) The total sample size is 100 plaques (50 per group) based on what is feasible for recruitment. (3) Assume that 20% of subjects will have both asymptomatic and symptomatic stenosis, so these subjects will contribute to both groups. (4) In adjusting for the correlation that is introduced by including subjects in both the asymptomatic and symptomatic arms of the study, the ICC is assumed to be between 0.2 and 0.4. (5) Assume a two‐sided test.

Based on the data summarized in Table 2, the following estimated frequencies were used for each of the plaque components: (1) intraplaque hemorrhage: 46% in symptomatic and 14% in asymptomatic; (2) thin or ruptured fibrous cap: 68% in symptomatic and 32% in asymptomatic; (3) large lipid core area: 75% of symptomatic score > 1 and 40% of asymptomatic are scored > 1.

The estimated power for testing each plaque component was determined using the estimated frequency of each component as follows: intraplaque hemorrhage: if the intraclass correlation coefficient (ICC) = 0.2 the power is 82%, if the ICC = 0.3 power is 80%, and if the ICC = 0.4 the power is 79%; thin or ruptured fibrous cap: if the ICC = 0.2 the power is 84%, if the ICC = 0.3 power is 83%, and if the ICC = 0.4 the power is 82%; and lipid Core Score: if the ICC = 0.2 the power is 82%, if the ICC = 0.3 the power is 81%, and if the ICC = 0.4 the power is 80%.

For these analyses, both unadjusted and adjusted models will be implemented for each of the three potential high‐risk plaque features. A logistic regression model adjusted for the propensity score (D'Agostino 1998), due to the nonrandom selection of lesions and other covariates of interest that could influence the presence of these atherosclerotic features, will be used. The results are expected to show that the frequency of at least one of the plaque components is significantly higher in the symptomatic plaques than the asymptomatic plaques.

## Conclusion

Characterization of intracranial atherosclerotic stenosis using high‐resolution MRI is a prospective observational study that aims to demonstrate high‐interrater agreement for identifying intracranial plaque components on HR MRI, determine the frequency of these components in symptomatic versus asymptomatic plaques, and estimate the 1‐year rate of stroke in the territory of high‐risk plaque components. The results will inform the design of future multicenter studies to determine the prevalence and prognosis of intracranial atherosclerotic plaque components. Such studies could lead to new understanding of the pathophysiological mechanisms of cerebral ischemia in patients with atherosclerotic intracranial stenosis, improvements in risk stratification, and potentially to new treatments of this common and serious disease.

## Conflict of Interest

TNT – receives funding from NIH (K23 NS069668) for HRMRI research. TL and RM receive salary support from NIH (K23 NS069668). ZR, MIC, SS, RJA, MVS, TRB – no conflicts of interest.
